# Radiofrequency ablation of varicose veins in combination with ultrasonic-assisted wound debridement and platelet-rich plasma as well as platelet-rich fibrin technologies in treatment of lower extremity venous ulcers in office-based surgery

**DOI:** 10.11604/pamj.2022.42.154.29834

**Published:** 2022-06-24

**Authors:** Volodymyr Goshchynsky, Yrij Svidersky, Bogdan Migenko, Oleg Pyatnychka

**Affiliations:** 1 Department of Surgery, Institute of Postgraduate Education, I. Horbachevsky Ternopil National Medical University, Ternopil, Ukraine,; 2 Department of Internal Medicine N^o^ 2, I. Horbachevsky Ternopil National Medical University, Ternopil, Ukraine

**Keywords:** Ultrasonic debridement, venous ulcers, platelet-rich plasma, radiofrequency ablation

## Abstract

**Introduction:**

for the treatment of varicose ulcer performed clinical and laboratory reasoning of the use of ultrasonic-assisted venous ulcer debridement and Platelet-rich plasma with radiofrequency ablation in an outpatient setting, was carried out.

**Methods:**

ultrasonic-assisted debridement of trophic ulcer were performed for 50 patients with lower extremity varicose veins at decompensation stage. The effectiveness of ultrasonic-assisted debridement was evaluated by indicators of bacteriological, morphological, cytological study and assessment of trophic ulcers according to the MEASURE system. After ultrasonic-assisted debridement, the patients were divided into two groups: 30 patients, who underwent combined Platelet-rich plasma to stimulate wound regeneration and 20 patients, for whom the Granuflex hydrocolloid bandage was applied for the same purpose.

**Results:**

a comparative analysis of ulcer regeneration in two groups of patients proved that in cases of platelet rich plasma the time of transition from inflammatory-regenerative type to regenerative one is much shorter than when using a hydrocolloid dressing. In 28 patients undergoing Platelet-rich plasma (PRP) and Platelet-rich fibrin (PRF), the radiofrequency ablation of the principal superficial and perforating veins was performed. Another 22 patients performed autodermoplasty of trophic ulcers after radiofrequency ablation.

**Conclusion:**

our experience has shown that in a one-day inpatient surgical clinic such a multidisciplinary approach to treatment of venous ulcers, including ultrasonic-assisted debridement that is stimulation of wounded process by Platelet-rich plasma with further surgeries to remove the causes of decompensated chronic insufficiency, is promising regarding low costs of treatment and rehabilitation of these patients.

## Introduction

Between 600,000 and 2.5 million people worldwide suffer from chronic trophic ulcers of the feet and lower legs. On average, according to the literature, venous ulcers or ulcers, which are also called venous stasis ulcers (VSU), are 70-75% of all ulcers of the lower extremities and occur in 2% of the adult population of highly developed countries. In elderly and senior patients, the incidence increases in more than 3 times and ranges 4-6%. The development of these ulcers is caused by progression of chronic venous insufficiency, in particular in cases of lower extremity varicose vein disease (LEVVD) [1-3]. It should be noted that for patients with decompensated chronic venous insufficiency the costs of treatment is significantly higher, social activity reduces and their disability incidence increases. Thus, venous ulcers are an urgent medical and socio-economic issue. This requires search for new integrated technologies in the treatment of patients with LEVVD and trophic ulcers. In this regard, the use of radiofrequency vein ablation for “prepared” trophic ulcers for this is promising. Currently, there are many traditional and new technologies for cleansing ulcers from necrotic tissues followed by acceleration of their regeneration [4,5]. The treatment of these ulcers is based on a contemporary strategy of wound bed preparation [6]. Its main purpose is to accelerate wound healing. To achieve this, the concept of TIME (T-tissue, I-infection, M- moisture balance, E - epithelialization) is developed, which provides first of all the protection from negative factors that delay regeneration in the wound and its subsequent stimulation [3]. Debridement is crucial in this concept that is cleansing of ulcers from necrotic and devitalised tissues; it can be carried out by mechanical (surgically), autolytic, biological (maggot therapy), physical (low frequency ultrasonic cavitation, shock wave therapy, cryodestruction) means [5,7-10]. Our experience in the treatment of venous ulcers has shown that only surgical removal of their causes can successfully solve this problem. At the same time, operations on superficial varicose and perforating veins in cases of infected trophic ulcer in most cases lead to purulent-inflammatory complications. In our opinion, the use of ultrasonic-assisted debridement for cleansing of trophic ulcers from necrotic tissues, as an initial stage for the subsequent stages of treatment of decompensated form of LEVVD followed by RFA and simultaneous stimulation of regeneration in it by PRP and PRF therapy, is a promising method of treatment of this pathology in office-based surgery. It is quite attractive because of accessibility, significantly lower costs and fast social rehabilitation. The aim of the study is to provide a clinical reasoning for effectiveness of ultrasonic-assisted debridement in combination with radiofrequency vein ablation and PRP, PRF technologies for treatment of lower extremity varicose veins complicated by trophic ulcers in office-based surgery.

## Methods

**Study population and data collection:** fifty patients with lower extremity varicose veins at the stage of decompensation (C6 according to the CEAR classification) were followed up in the ambulatory surgery centres in Zhytomyr, Ternopil and in the City Phlebological Centre at the Department of Surgery of the Faculty of Postgraduate Education of Ternopil National Medical University (from the Ukraine). There were 29 females, 21 male patients; the age of the patients was 55±4.6 years old. The size of trophic ulcers ranged from 2.5×1.5 cm to 8.5×7.5 cm, the disease duration was 1.1±06 years that was considered chronic. The average size of ulcers was 4.5±1.7 cm. Criteria of inclusion were the presence of superficial or partial venous trophic ulcers; the wound should have corresponded to the first phase of wound process. Exclusion criteria were deep trophic ulcers, the bottom of which was muscles, fascia, bones; bone and shoulder index ≥ 0.7; surgical treatment of ulcers, skin infections, occlusive diseases of the lower extremities, diabetes mellitus; uncompensated heart failure; systemic connective tissue diseases; cancer; peripheral nerve damage, hormone therapy; lymphedema; pregnancy; other diseases at the stage of decompensation. In all cases, trophic ulcer corresponded to the first phase of the wound process (fibrin, detritus and purulent-necrotic masses at the bottom of the ulcer).

**Instrumental and laboratory studies:** all patients underwent ultrasound diagnosis to establish phlebohemodynamic disorders in the limb. Ultrasound examination of the venous system of the lower extremities was performed by means of the Vivid 3 device (General Electric, USA) with a sensor frequency of 5-10 MHz and the appropriate standard software package produced by the same company for the study of the venous system. The patients were examined in the afternoon in a vertical and horizontal position. The blood flow in the veins, diameters and shapes of the veins lumen, their deformation and sac-like transformation, wall thickness, homogeneity, elasticity of the valves, their function at hydrostatic loading tests, presence of blood reflux, duration of retrograde flow through the veins and its circulation to anatomic segments, a state of sapheno-femoral and sapheno-popliteal mouth were determined by ultrasound examination. Attention was paid to localization of perforating veins, determining their diameter and duration of venous blood reflux in them. In all patients trophic ulcers were evaluated before applying an ultrasonic debridement according to the MEASURE system. It involved measurement of length, width, depth, area of the ulcer, the amount of exudate and studying its profile, the pattern of the wound bed, pain, the presence or absence of tissue necrosis, the state of the wound edges and surrounding tissues. For dynamic registration of planimetric parameters of wound defect healing, the mobile application +WoundDesk was used, which was based on an Android smartphone camera. During the photography, the indicator scale “+WD” was used, which had been added by the authors of the application; it provided determination of trophic ulcer contours. Dynamic bacteriological examination of the trophic ulcers content was performed during the initial examination of the patient as well as after the procedure of ultrasonic-assisted debridement. Biological material was collected using sterile swabs and placed in the Amies transport medium. Inoculation of ulcer discharge was performed by the method of sequestration using dense nutrient media. The isolated strains were identified by means of the “miniApi” semi-automatic analyzer (BioMerieux, France). Preparations for cytological examination were prepared in this way: sterile degreased slides were applied to the center of the trophic ulcer and fixed by drying in air then stained with azure-eosin according to Romanovsky for 3 minutes, differentiated in distilled water and dried. The obtained preparations were studied in a biological microscope at a magnification of x40, and time photographed at the same using a Micro webcam. The results were evaluated by the number of cells per area unit, by the state of cell types. Also, to determine the profile of the wound in the ulcer, a biopsy of its top layer was performed. The resulting material was transferred to a glass slide, fixed with 96% ethyl alcohol for 2 minutes and stained by the method of Romanovsky-Gimza (for 15 minutes). The analysis of the relative content of the following cells was studied in these preparations: segmental neutrophils, stab neutrophils, phagocytic neutrophils, degenerative neutrophils, eosinophils, lymphocytes, monocytes, histiocytes, macrophages, fibrocytes, fibroblasts, endothelium. The result was expressed in percents per 100 cells counted.

**The ultrasonic-assisted debridement:** the first stage was preparation for surgery; all patients underwent ultrasonic cavitation of trophic ulcers with the SONOCA 180^®^: device (produced by SÖRING Gmb Company (25 kHz)). The required power of impact on the wound surface was set on the control panel of the device depending on the array of devitalized tissues or purulent-fibrinous layers. The frequency of use of the ultrasound machine was 2 procedures with a 2-3 day interval. 0.9% saline was used as an irrigation fluid, in which to enhance decontamination a prontosan solution was added in a ratio of 1:10. Ultrasound treatment of the ulcer was performed under local anesthesia with a 0.75% solution of ropivocaine as an anesthetic. It should be noted that tips of different shapes were used that allowed their adapting to the trophic ulcer surface. Thus, in the treatment of deep ulcers of complex configuration a “ball” was used, and in simpler relief trophic ulcers -a “hoof”. The duration of exposure to the wound was 5-15 seconds per 1 cm^2^ of wound surface. Systemic antibacterial therapy was not performed. The patient underwent 2 sessions of ultrasound ulcer treatment: the first session on the third day after examination of the patient, the second session-immediately before radiofrequency vein ablation and PRP as well as flexible coating of ulcers with PRF (plasma membrane). After ultrasonic-assisted debridement, patients were divided into two groups: the 1^st^ group involved 30 patients, for whom a combination of PRP and PRF was applied to stimulate the wound regeneration; the 2^nd^ group- 20 patients, for whom the Granuflex hydrocolloid bandage (produced by Conva Tec-England) was applied for the same purpose.

Methods of performing manipulations with Plasma Rich Growth Factor: Plasma Rich Growth Factor (PRGF^®^-ENDORET^®^) was prepared according to the protocol of the Institute of Biotechnology BTI (Spain). 36 ml of blood in 4 tubes of a volume of 9 ml containing 3.8% sodium citrate was taken in the patients. Blood was centrifuged (BTI System IV centrifuge) at a rate of 580 g for 8 minutes; the obtained plasma was separated into fraction (F1) and fraction (F2). A 2 ml fraction of F2 was taken from each tube for injection. Then, at an aseptic manipulation, subcutaneous injections on the periphery of the trophic ulcer were performed, departing up to 1 cm from the edge of the ulcer and 2 cm space between injection sites. At the same time, the ulcer surface was covered with a plasma rich growth factors membrane, which was prepared using F2. Depending on the area of the ulcer, one or two plasma membranes were used. To reduce the vertical discharge RFA was used according to the method of VNUS- Closure FAST by a high-tech device COVIDIEN (prodused by Medtronics Company). Ethical considerations: Ukrainian Committee on Bioethics and Deontology reviewed and approved the study. Also, these studies were approved by the Bioethics Commission of Ternopil National Medical University. Statistical analysis: statistical data was processed using the licensed electronic analysis packages “STATISTICA 10.0” and “Microsoft Excel”. The calculations were performed by the methods of parametric statistics with calculation of the arithmetic mean and standard deviation. Data are presented as M±m standard deviation (m). Also, the Student´s t-test was used to determine the statistical significance (at p<0.05)

## Results

Before treatment after microbiological examination of trophic ulcers the growth of the following microorganisms was revealed: *E. coli + P. aeruginosa, P. aeruginosa + S. aureus, P. aeruginosa, S. aureus, S. epidermidis, P. aeruginosa + E. coli, S. epidermidis, S. aureus + P. aeruginosa, S. aureus + E. coli, P. aeruginosa, S. aureus + P. aeruginosa, Acinetobacter sp.*, Klebsiella, with an initial level of bacterial contamination of 105-108 microbial bodies in 1 g of tissue. In 7 days after ultrasonic cavitation, mainly *Pseudomonas aeruginosa, Staphylococcus aureus, Staphylococcus epidermidis* were plated, and in 29% and 21% of cases - non-fermenting gram-negative bacteria and enterobacteria, respectively, but the level of bacterial contamination in tissues decreased to 102-103 microbial bodies in 1 g of tissue. Analysis of cytograms of the patients with wound ultrasonic cavitation proved their inflammatory-regenerative type (Table 1). Thus, in the cytogram neutrophils dominated, mainly owing to segmental neutrophils. Phagocytic neutrophils (10.5±1.1%) and degenerative neutrophils (7.2±0.6%) were also detected. The degenerative neutrophils swelled and had indistinct contours with a changed shape of the nuclei and a red tinge. Neutrophil phagocytes were large and had vacuoles and inclusions in the cytoplasm. There was a small number of macrophages and fibroblasts, 1.4±0.5 and 0.8±0.3%, respectively (Table 1).

**Table 1:** cytogram of trophic ulcers before and after ultrasonic-assisted debridement

Variables	Content of cells in trophic ulcer, % (M±m)		
Cell type	Before ultrasonic-assisted debridement	After ultrasonic-assisted debridement	After a second time ultrasonic-assisted debridement
**Segmental neutrophils**	46.0±2.9	21.0±1.4	19.3±1.2
**Stab neutrophils**	4.6±0.1	3.3±0.2	2.4±0.3
**Phagocytic neutrophils**	10.5±1.1	6.2±1.2	6.0±1.1
**Degenerative neutrophils**	7.2±0.6	6.5±0.6	7.0±0.7
**Eosinophils**	2.2±0.3	2.0±0.2	0.25±0.1
**Lymphocytes**	21.7±0.6	16.2±0.3	16.7±0.4
**Monocytes**	2.0±0.3	3.0±0.3	3.0±0.6
**Histiocytes**	3.9±0.3	5.0±1.2	3.5±0.9
**Macrophages**	1.4±0.5	5.2±0.3	7.0±0.4
**Fibroblasts**	0.8±0.3	8.5±0.4	10.0±0.7
**Fibrocytes**	-	21.0±2.0	24.0±2.9
**Endothelium**	-	+	+
**Epithelium-**	-	2.0±0.2	0.25±0.1
**Type of cytograms**	Inflammatory-regenerative	Regenerative-inflammatory	Regenerative-inflammatory

**%:** percentage; **(M±m):** Means±SD

**Figure 1:**
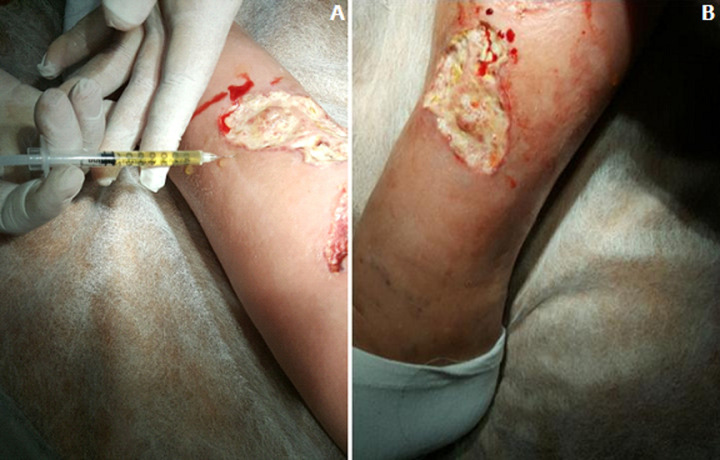
A,B) patient S, plasma injection to the ulcer periphery

**Figure 2:**
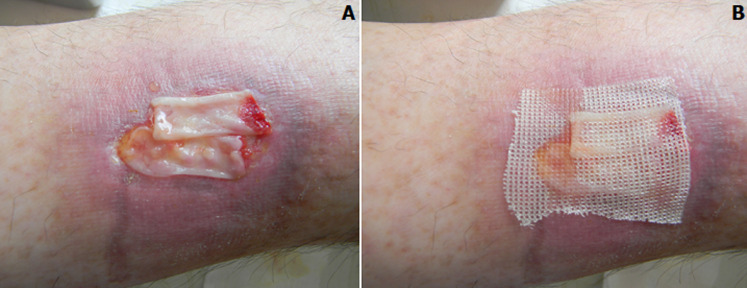
A,B) patient S, closing a trophic ulcer surface with a plasma membrane

After the first session of ultrasonic-assisted debridement the wounds were of a regenerative-inflammatory type. A decrease in the number of segmental neutrophils (up to 21.0±1.4) and degenerative neutrophils (up to 6.5±0.6) was evidenced with a total number of neutrophilic cell elements of less than 53.1%. An increase in connective tissue cells was also established; some of them were fibroblasts (up to 8.5 ± 0.4); the number of histiocytes (up to 5.0 ± 1.2) and macrophages (up to 5.2 ± 0.3) increased as well. This cell composition evidenced the transition to wound cleansing from necrotic tissues, as their function is to absorb the products of cell breakdown. After the second session of ultrasonic-assisted debridement, the total number of neutrophils of different types decreased significantly. In the smears the connective tissue cells (fibrocytes and fibroblasts) dominated. Endothelial and squamous epithelial cells also were present diffusely or clustered. Thus, ultrasonic-assisted debridement promotes further stimulation of regeneration in trophic ulcers (Figure 1, Figure 2).

It was established that after the first session of PRP and PRF (Figure 1, Figure 2) the patients had less pain from (5.0±0.2) to (1.4±0.6) points in the trophic ulcer region, normal body temperature, sleep and appetite. Also in these patients compare to the control group, the area and depth of wound defects decreased by 23.1%, 37.4% and 79.9% on the 5^th^-6^th^, 8^th^-9^th^, 12^th^-14^th^ day, respectively, compare to the initial data. Decreased perifocal oedema and redness of the skin around the wound was evidenced in most patients already on the 3^rd^ day of PRP and PRF therapy. It was found out that after the second session of PRP and PRF (in 4-5 days) the average time of tissue granulation in these patients was on the (6.74 ± 1.65) day, and the beginning of marginal epithelialization-on the (6.2±1.44) day; at the same time in the control group of patients these processes developed more slowly-on the (9.73±1.4) and (11.25±1.7) days, respectively (p < 0.05). The results of cytological studies showed that the above methods led to a decrease in the number of cells in the wound smears, which determine the acute phase of inflammation (neutrophils, lymphocytes, monocytes), and an increase the number of cells responsible for reparative processes (macrophages, fibroblasts). Thus, in the patients a decrease in the content of neutrophils and lymphocytes was evidenced on the 2^nd^-3^rd^ day, on the 4^th^-5^th^ day- of all inflammatory cells. Dendrites, microbial bodies disappeared. Significant epithelialization was a characteristic feature. This proved the transition from regenerative-inflammatory to regenerative type of ulcer healing.

**Figure 3:**
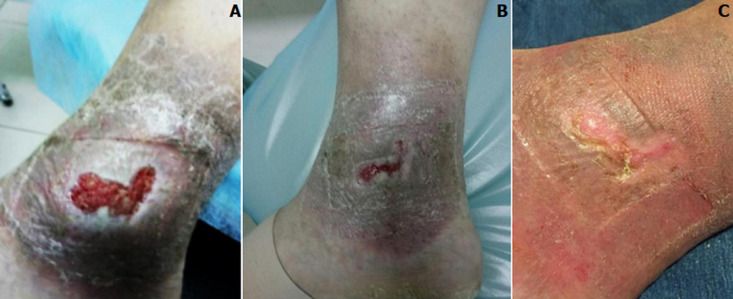
A,B) trophic ulcer after PRP and PRF therapy; C) ulcer healing after RFA

This trophic ulcer state allowed performing radiofrequency ablation of the main superficial and perforating veins simultaneously with PRP and PRF in 28 patients (Figure 3). The severity of venous disease according to the VCSS scale in the main group decreased from (12 ± 0.2) points to (5 ± 0.3); at the same time in the control group this parameter was (8±0.4). It should be noted that the average day of pre-surgery preparation of patients with trophic ulcers with PRP and PRF application was (7.8 ± 4.6) days, contrary to (14.6±3.7) days in the control group. This made a complete RFA of varicose veins possible as well as additional elimination of horizontal reflux in the area of trophic changes by RFA of incapable perforating veins with ultrasound guided navigation.

## Discussion

The number of patients with lower extremity venous ulcers is increasing continually. Significant costs is required for treatment and rehabilitation of these patients [1,11,12]. A huge variety of trophic ulcer treatment methods indicates the lack of a single reliable therapy that allows getting a lasting positive result. It is clear that the main stage that removes the pathophysiological causes of trophic ulcers is correction of vertical and horizontal reflux in the system of the great and small veins as well as in the perforating veins [4,13]. However, the presence of “infected” trophic ulcers deters surgeons from surgical treatment due to a large number of possible purulent-inflammatory complications. In this regard, an effective treatment of trophic ulcers in the preoperative stage is the priority. The current strategy of chronic wounds treatment, as well as of trophic ulcers (wound bed preparation) [14], is a comprehensive intervention in the wound course, the result of which is the formation of healthy granulation tissue. Thus, the developed concept of TIME (T-tissue, I-infection, M-moisture balance, E-epithelialization) provides a comprehensive impact on local and systemic factors that directly affect the regeneration of tissues in the ulcer. According to the literature, the main link in the concept of TIME at the beginning of trophic ulcer treatment is restoration of functions and state of tissues that fill the bed with debridement; it allows cleansing the wound from necrotic and devitalised tissues [15,16-19].

However, the debridement methods are quite uncertain. Which method should be used: surgical, ultrasonic-assisted, cryodestruction, hydrosurgical, biodebridation, enzymatic, laser, plasma necrectomy or maggot therapy [20-23]. The lack of objective assessment of the effectiveness of any debridement method somewhat constrains its use for trophic ulcer treatment, especially in an outpatient setting [9-11]. In our opinion, ultrasonic-assisted debridement of trophic ulcers, after a comprehensive assessment of its use in ulcer wound course, can be a significant step towards a final surgical treatment of lower extremity varicose veins. In recent years, much clinical interest is paid to a growth factor therapy released from activated platelets during whole blood centrifugation as an alternative to other methods of influence on the regeneration in trophic ulcers [23]. Conventional treatment, such as dressings, surgery, and even skin grafts, are not responsible for adequate healing, as these methods cannot provide the substances for healing modulation [24]. In 1986, Knighton *et al*. [25] showed that accelerated epithelialization of granulation tissue leading to full recovery of chronic non-healing ulcers was achieved using autologous platelet factors. This was the first clinical prove that locally acting factors derived from autologous blood promote healing of chronic skin ulcers. In this study, the time to 100% healing after initiation of platelet healing factors (PDWHF) was 7.5±6.5 weeks. There was a direct correlation between the start of PDWHF therapy and 100% healing. The age of the patients and the location of the ulcers did not have any statistically significant effect on PDWHF-stimulated wound healing.

It has been proved that platelet-rich plasma improves wound healing by promoting a healing process secondary to its GF. These include platelet-derived GF (ÞÞ,ßß, and Þß), fibroblast GF, vascular endothelial GF, epidermal GF, insulin-like GF, and transforming GF. These GFs stimulate mesenchymal cell recruitment, proliferation, extracellular matrix degeneration, and cell differentiation for tissue regeneration. These factors are released from Þ-granules in response to platelet activation by platelet aggregation inducers [26,27]. In addition to GF, platelets release other numerous substances (e.g. fibronectin, vitronectin, and sphingosine 1-phosphate) that are important for wound healing. Moreover, the advantage of PRP over a single recombinant delivery of human GF is the release of multiple GFs and differentiation of factors in platelet activation [28]. According to several studies, most trophic ulcers responded by acceleration of regenerative process in the wound and a significant reduction of its area [29-36]. It was found out in our study that PRP and PRF are an effective, simple and reasonably priced method of chronic lower extremity ulcers treatment. However, some more controlled randomized prospective clinical trials are needed to prove its effectiveness definitively. There is also a necessity to develop a standard protocol for preparation of PRP, as currently in the literature there is no standardised procedure for its application.

## Conclusion

Our experience has shown that in a one day inpatient surgical clinic such a multidisciplinary approach to treatment of venous ulcers, including ultrasonic-assisted debridement that is stimulation of wounded process by PRP and PRF with further surgeries to remove the causes of decompensated chronic insufficiency, is promising regarding low costs of treatment and rehabilitation of these patients.

### What is known about this topic

- The treatment of trophic ulcers is a modern strategy - wound bed preparation; the main goal of this strategy is accelerating the wound healing process; to achieve this goal was developed the concept of TIME (T-tissue, I-infection, M-moisture balance, E-epiteliseition), which involves, first of all, the elimination of negative factors that delay the regeneration process in the wound and its subsequent stimulation;
- The main role in the concept of TIME is given to debridement- purification ulcers from necrotic and non-viable tissues, which can carried out mechanical (surgical), autolytic, biological (larvae of flies), physical (low frequency ultrasonic cavitation, shock wave therapy, cryodestruction);
- Among the debrimentation of wounds, their ultrasonic cavitation is considered one of the effective methods.

### What this study adds

- Through the use of microbiological, cytological and morphological research methods we found that indeed, ultrasonic debrimation of venous trophic ulcers translates chronic inflammatory process in acute, and thus accelerates the process regeneration in them; this method is effective in the treatment of venous trophic ulcers;
- We have proposed a phased preparation of patients with varicose veins lower extremity disease complicated by venous trophic ulcer before surgery in an outpatient setting, including ultrasound ulcer debridement-stimulation of the regeneration process with PRP and PRF-radiofrequency ablation of the great saphenous vein and perforating veins, if necessary with autodermal plastics of trophic ulcers;
- Similar stages in the treatment of this category of outpatients conditions, reduces the time of preparation of patients for surgical treatment underlying pathology (varicose veins) and reduces material costs for their treatment and rehabilitation.
